# Association between oxidative balance score and chronic obstructive pulmonary disease: A cross-sectional study

**DOI:** 10.1097/MD.0000000000039883

**Published:** 2024-10-04

**Authors:** Weiyan Chen, Wei Zhang

**Affiliations:** a Guangzhou University of Chinese Medicine, Guangzhou, Guangdong, China; b The First Affiliated Hospital of Guangzhou University of Chinese Medicine, Guangzhou, Guangdong, China.

**Keywords:** chronic obstructive pulmonary disease, dietary pattern, lifestyle, oxidative balance score, oxidative stress

## Abstract

Oxidative stress is an essential contributor to the progression of chronic obstructive pulmonary disease (COPD). A systematic assessment of diet patterns and lifestyle with the oxidative balance score (OBS) to reflect oxidative stress levels will help predict the risk of COPD. This study conducted a cross-sectional analysis to assess the link between OBS and COPD. 5162 participants were collected from 2013 to 2018 from the National Health and Nutrition Examination Survey (NHANES). Multivariate logistic regression models were applied to assess the relationship between OBS and COPD prevalence. The linearity of the association was explored using smoothed curve fitting. In addition, further subgroup analysis and interaction tests were conducted to ascertain the consistency of the relationship across diverse populations. Results of the multivariate logistic regression models indicated a negative association between OBS and the odds of COPD prevalence. Each incremental unit in OBS correlated with a 3% reduction in the odds of COPD in the fully adjusted model (OR 0.97, 95% CI 0.95–0.99). Further analysis by OBS tertiles indicated that individuals in the highest OBS tertile (T3) had a 17% lower probability of COPD compared to those in the lowest tertile (T1) in the fully adjusted model (OR 0.83, 95% CI 0.64–0.97). The smoothed curve fitting supported the negative association between OBS and COPD. Subgroup analyses revealed that the protective effect of OBS was notably pronounced among the non-hypertensive and non-diabetic populations. These findings suggest a negative link between OBS and COPD, underscoring the importance of antioxidant-rich diets and lifestyles in preventing COPD.

## 1. Introduction

Chronic obstructive pulmonary disease (COPD) is a prevalent respiratory disease characterized by cough, sputum production, and persistent airflow limitation.^[1]^ COPD affects over 380 million individuals worldwide and has emerged as the third leading cause of death globally.^[2]^ COPD is linked to increased usage of healthcare services, diminished quality of life, and higher rates of mortality.^[3,4]^ However, the pathogenesis of COPD remains unclear. Air pollution exposure is considered an important risk factor for COPD, and genetic susceptibility and lifestyle are also involved in development of COPD.^[5]^ More and more evidence suggest that oxidative stress caused by lifestyle and dietary habits may be an essential factor affecting the incidence of COPD.^[6]^ However, the specific association among them is still unclear.

Oxidative stress, which arises due to an imbalance in the production of reactive oxygen species and the body’s defense mechanisms against them, is a significant pathogenic factor in COPD.^[7]^ Oxidative stress may promote the development of COPD by damaging cellular DNA, decreasing antiprotease activity, and accelerating small airway fibrosis.^[8]^ A healthy lifestyle, such as no smoking and no drinking alcohol, is considered closely related to the prevention and good prognosis of various diseases. Smoking is widely considered a significant factor in COPD,^[9]^ followed by obesity, alcohol consumption, and sedentary behavior. A healthy lifestyle enhances antioxidant protection, reduces oxidative stress,^[10–12]^ and potentially reduces the risk of chronic obstructive pulmonary disease such as COPD.^[13]^

In addition, a healthy dietary pattern, such as high dietary fiber, vitamins, and minerals, is considered beneficial for physical health.^[14,15]^ Perhaps it is because these substances are rich in antioxidants, which can affect COPD’s occurrence and development by regulating the levels of inflammatory factors.^[16]^ Antioxidants in the process of oxidative stress. Western diet patterns are characterized by higher consumption of antioxidant foods, which may be one of the reasons why it has become a region with a high incidence rate of COPD. Epidemiological evidence suggests that consuming antioxidant-rich foods in adulthood is related to reduced risk and better prognosis of COPD.^[17–19]^

The oxidative balance score (OBS) is often used to evaluate the exposure to anti-oxidants and prooxidants in diet and lifestyle, reflecting the overall burden of oxidative stress.^[20]^ A higher OBS indicates more exposure to antioxidants and less exposure to prooxidants. Previous studies have shown that OBS is inversely proportional to the incidence rate of various chronic diseases such as depression,^[21]^ diabetes,^[22]^ cardiovascular disease^[23]^ and periodontitis.^[24]^ However, the relationship between OBS and COPD has not yet been explored.

Therefore, this study aims to systematically evaluate the correlation between OBS and COPD prevalence using information from the National Health and Nutrition Survey (NHANES), providing a theoretical reference for preventing COPD by changing diet and lifestyle.

## 2. Methods

### 2.1. Study population

**Figure 1.**
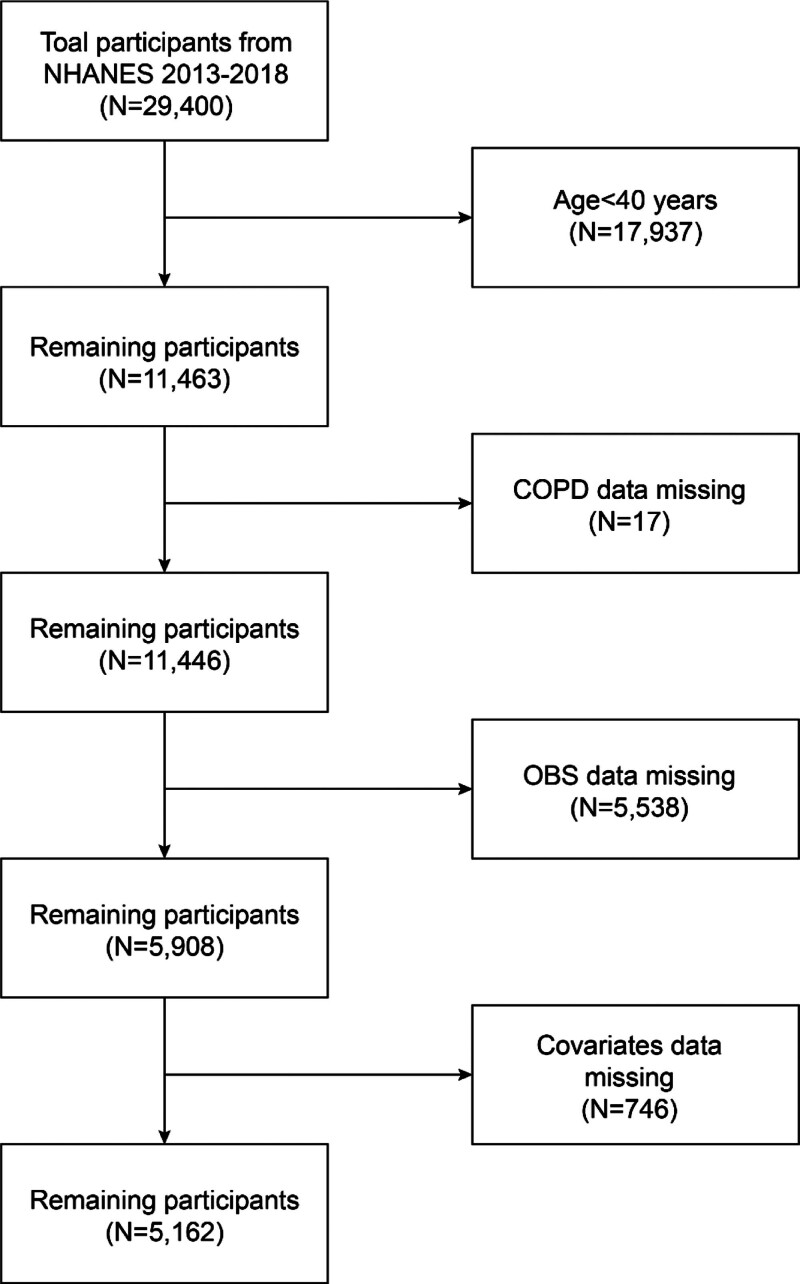
Flowchart of study population included in analysis. COPD = chronic obstructive pulmonary disease, NHANES = National Health and Nutrition Examination Survey, OBS = oxidative balance score.

NHANES is a cross-sectional survey conducted by the US National Center for Health Statistics of Centers for Disease Control and Prevention. It aims to assess American civilian’s health and nutritional status through interviews, physical examinations, and lab tests.^[25]^ The Research Ethics Review Board of the National Center authorized and approved NHANES, with all participants providing written informed consent before participating. Data for this study was obtained from NHANES 2013 to 2018, with 5162 subjects selected after excluding certain criteria. The flow chart for participant selection is detailed in Figure 1.

### 2.2. Assessment of COPD

COPD was defined based on responses to the question, “Has a doctor or other health professional ever told you that you have COPD?.” A positive response to this question identified individuals as COPD patients.^[26,27]^

### 2.3. Measurement of OBS

Previous research has thoroughly explained the establishment and calculation of OBS.^[28]^ OBS consists of 16 dietary components and 4 lifestyle factors that were evaluated for their impact on oxidative stress. These factors can be classified as either prooxidative (such as alcohol consumption, total fat, iron, BMI, and smoking) or anti-oxidative (such as dietary fiber, beta-carotene, riboflavin, niacin, vitamin B6, total folate, vitamin B12, vitamin C, vitamin E, calcium, magnesium, zinc, copper, selenium, and physical activity). Participants were classified based on alcohol consumption levels as nondrinkers, non-heavy drinkers (0–15 g/d for females and 0–30 g/d for males), and heavy drinkers (≥15 g/d for females and ≥ 30 g/d for males) and were assigned scores of 2, 1, and 0 respectively. Other components were divided into tertiles according to gender. The antioxidant group received a score of 0 to 2 from tertile 1 to tertile 3, whereas the pro-oxidant group received a score of 2 to 0 from tertile 1 to tertile 3. The sum of scores for each component was used to calculate the total OBS score. Table 1 describes the detailed allocation criteria for OBS components.

**Table 1:** Oxidative balance score assignment scheme.

OBS components	Male			Female		
	0	1	2	0	1	2
Dietary fiber (g/d)	<13.6	<21.65	≥21.65	<11.71	<18.30	≥18.30
β-carotene (RE/d)	<676.44	<2204.16	≥2204.16	<730.84	<2457.73	≥2457.73
Vitamin B2 (mg/d)	<1.72	<2.51	≥2.51	<1.35	<1.96	≥1.96
Niacin (mg/d)	<21.84	<31.38	≥31.38	<15.93	<22.72	≥22.72
Vitamin B6 (mg/d)	<1.71	<2.53	≥2.53	<1.29	<1.90	≥1.90
Total folate (mcg/d)	<311	<474	≥474	<251.5	<378.5	≥378.5
Vitamin B12 (mcg/d)	<3.32	<5.81	≥5.81	<2.37	<4.09	≥4.09
Vitamin C (mg/d)	<41.39	<100.35	≥100.35	<41.65	<94.13	≥94.13
Vitamin E (mg/d)	<6.55	<10.28	≥10.28	<5.62	<8.98	≥8.98
Calcium (mg/d)	<726.89	<1101.11	≥1101.11	<596	<917.88	≥917.88
Magnesium (mg/d)	<262	<369.5	≥369.5	<214.5	<301.5	≥301.5
Zinc (mg/d)	<9.34	<13.67	≥13.67	<6.91	<10.06	≥10.06
Copper (mg/d)	<1.00	<1.43	≥1.43	<1	<2	≥2
Selenium (mcg/d)	<100.75	<143	≥143	<73.66	<107.20	≥107.20
Total fat (g/d)	≥101.39	<101.39	<68.92	≥77.71	<77.71	<53.05
Iron (mg/d)	≥17.43	<17.43	<11.88	≥13.54	<13.54	<9.31
Physical activity (MET, min/wk)	<360	<1280	≥1280	<240	<720	≥720
Alcohol (g/d)	≥30	<30	None	≥15	<15	None
Body mass index (kg/m^2^)	≥30.6	<30.6	<26.2	≥32.1	<32.1	<26.1
Cotinine (ng/mL)	≥0.196	<0.196	<0.011	≥0.048	<0.048	<0.011

MET = metabolic equivalent, OBS = oxidative balance score.

Dietary nutrient intake and alcohol consumption were determined from two 24-hour dietary recall interviews. BMI was defined as weight in kilograms divided by the square of height in meters. Based on previous studies, serum cotinine was used as a proxy for smoking because it combines active and passive smoking levels. Physical activity was quantified using metabolic equivalent scores derived from physical activity questionnaires.

### 2.4. Covariates

In order to control for potential confounding variables, a number of closely related covariates were chosen, such as age, gender (male, female), race (Mexican American, non-Hispanic White, non-Hispanic Black, and others), level of education (below high school, high school, and above high school), household poverty-to-income ratio (PIR, categorized as low income: <1.3; middle income: 1.3–3.5; and high income: ≥3.5), marital status (married/living with partner, never married, widowed/divorced/separated), covered by health insurance (yes, no), daily energy intake, presence of hypertension (yes, no), and presence of diabetes (yes, no). Covariate data was collected through a questionnaire and physical examination. Hypertension was defined as self-reported hypertension use of oral antihypertensive medications or having 3 or more measurements with a systolic blood pressure ≥ 140 mm Hg or diastolic blood pressure ≥ 90 mm Hg out of 4 measurements.^[29]^ Diabetes was characterized as either self-reported diabetes, the utilization of antidiabetic medicines or insulin, or having a glycated hemoglobin level exceeding 6.5%.^[30]^

### 2.5. Statistical analysis

Participants were categorized into 2 groups according to the presence of COPD. Two categories based on COPD presence were assigned to the participants. Population proportions and percentages were utilized to express categorical variables, which were then compared using chi-square tests. Mean, and standard deviation were employed to describe continuous variables, with *t* tests for normally distributed data or Mann–Whitney *U* tests for skewed distributions. Multivariate logistic regression models were utilized to assess the correlation between OBS and COPD prevalence, with adjustments made for potential covariates. Model 1 did not adjust for any potential confounders. Model 2 adjusted for age, gender, and race. Model 3 was further adjusted for education level, PIR, daily energy intake, hypertension, and diabetes. Additionally, a smoothed curve fitting analysis was conducted to explore potential nonlinear correlations between OBS and COPD. Subgroup analyses and interaction tests were performed to evaluate the association between OBS and COPD across various populations, stratified by age, gender, race, education level, and PIR. Statistical significance was determined by a 2-sided *P* value <.05. R software (version 4.2) and EmpowerStats (version 4.1) were utilized for the statistical analyses in this study.

## 3. Results

### 3.1. Baseline characteristics

The baseline characteristics of individuals recruited in the NHANES 2013 to 2018 cycles are described in Table 2. In total, 5162 participants were included, of whom the average age was 58.39 ± 11.64 years and 50.99% were male. Among them, 262 individuals were diagnosed with COPD, with an overall prevalence of 5.08%. Except for daily energy intake, significant differences in demographic and clinical features were noted between the COPD and non-COPD groups. Compared to individuals without COPD, patients with COPD generally exhibited characteristics such as being older, male, non-Hispanic White, current or former smokers, having lower levels of education and family income, fewer never-married, covered by health insurance and a higher prevalence of hypertension and diabetes. In addition, individuals with COPD exhibited lower OBS.

**Table 2:** Baseline characteristics of the study population according to chronic obstructive pulmonary disease.

Basic information	Overall (n = 5162)	COPD (n = 262)	Non-COPD (n = 4900)	*P* value
Age, SD (yr)	58.39 ± 11.64	64.87 ± 10.17	58.05 ± 11.61	<.001
Gender (%)				.037
Male	2632 (50.99%)	150 (57.25%)	2482 (50.65%)	
Female	2530 (49.01%)	112 (42.75%)	2418 (49.35%)	
Race (%)				<.001
Mexican American	640 (12.4%)	7 (2.67%)	633 (12.92%)	
Non-Hispanic White	2246 (43.51%)	188 (71.76%)	2058 (42.00%)	
Non-Hispanic Black	1082 (20.96%)	37 (14.12%)	1045 (21.33%)	
Others	1194 (23.13%)	30 (11.45%)	1164 (23.76%)	
Education level (%)				<.001
Less than high school	882 (17.09%)	54 (20.61%)	828 (16.90%)	
High school	1183 (22.91%)	94 (35.88%)	1089 (22.22%)	
More than high school	3097 (60.00%)	114 (43.51%)	2983 (60.88%)	
PIR (%)				<.001
<1.3	1316 (25.49%)	114 (43.51%)	1202 (24.53%)	
1.3–3.5	1954 (37.85%)	102 (38.93%)	1852 (37.80%)	
≥3.5	1892 (36.65%)	46 (17.56%)	1846 (37.67%)	
Marital status (%)				<.001
Married/living with partner	3562 (69.00%)	134 (51.15%)	3428 (69.95%)	
Never married	932 (18.06%)	26 (9.92%)	906 (18.50%)	
Widowed/divorced/separated	668 (12.94%)	102 (38.93%)	566 (11.55%)	
Insurance status (%)				<.001
Yes	4232 (81.98%)	243 (92.75%)	3989 (81.41%)	
No	930 (18.02%)	19 (7.25%)	911 (18.59%)	
Smoking status (%)				<.001
Current smoker	988 (19.14%)	117 (44.66%)	871 (17.77%)	
Former smoker	1187 (22.99%)	101 (38.55%)	1086 (22.17%)	
Never smoker	2987 (57.87%)	44 (16.79%)	2943 (60.06%)	
Hypertension (%)				<.001
Yes	2330 (45.14%)	165 (62.98%)	2165 (44.18%)	
No	2832 (54.86%)	97 (37.02%)	2735 (55.82%)	
Diabetes (%)				<.001
Yes	936 (18.13%)	73 (27.86%)	863 (17.61%)	
No	4226 (81.87%)	189 (72.14%)	4037 (82.39%)	
Energy intake, SD (kcal/d)	2013.04 ± 780.27	1990.08 ± 767.13	2014.27 ± 781.02	.625
OBS (SD)	20.09 ± 7.00	17.51 ± 6.53	20.22 ± 7.00	<.001

COPD = chronic obstructive pulmonary disease, OBS = oxidative balance score, PIR = poverty-to-income ratio, SD = standard deviation.

### 3.2. Association between OBS and COPD prevalence

**Figure 2.**
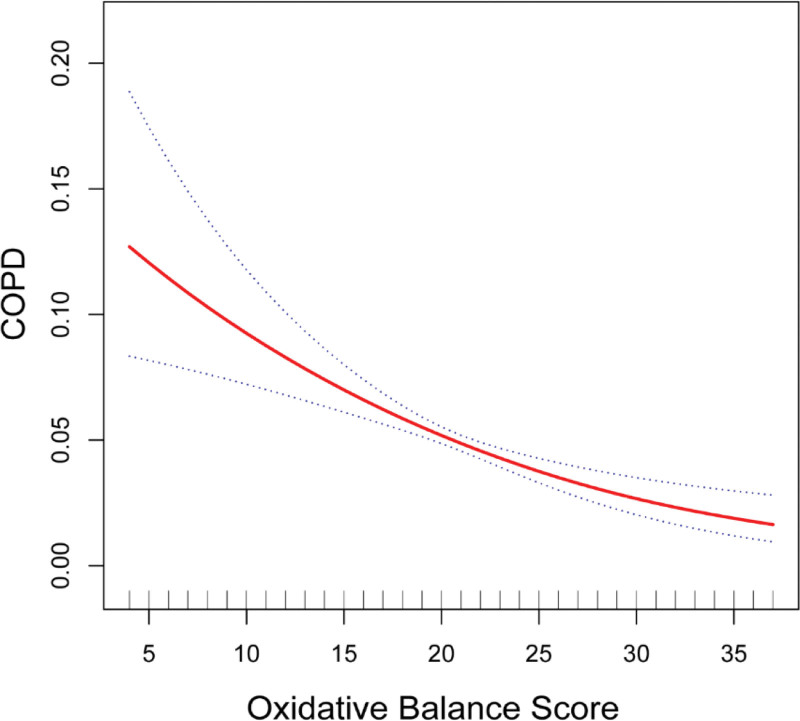
Correlation between Oxidative Balance Score and the odds of chronic obstructive pulmonary disease. The solid red line connecting the variables represented the smooth curve fit. The 95% confidence interval from the fitting was shown as blue bands. COPD = chronic obstructive pulmonary disease.

The study developed 3 models to assess the relationship between OBS and COPD prevalence, as depicted in Table 3. In each model, a significant adverse correlation between OBS and COPD prevalence was observed. A 1-unit rise in OBS was linked to a 3% decrease in COPD odds in the fully adjusted model (OR 0.97, 95% CI 0.95–0.99). Further analysis based on OBS tertiles revealed that individuals in the highest tertile (T3) had 17% lower odds of COPD compared to those in the lowest tertile (T1) in the fully adjusted model (OR 0.83, 95% CI 0.64–0.97). In addition, as shown in Figure 2, smooth curve fitting validated the inverse linear relationship between OBS and COPD prevalence (*P* for nonlinear = .15 > .05).

**Table 3:** Association between oxidative balance score and chronic obstructive pulmonary disease prevalence.

	Model 1		Model 2		Model 3	
	OR (95% CI)	*P* value	OR (95% CI)	*P* value	OR (95% CI)	*P* value
OBS	0.94 (0.93, 0.96)	<.0001	0.94 (0.92, 0.96)	<.0001	0.97 (0.95, 0.99)	<.0001
OBS tertiles						
Tertile 1	Reference		Reference		Reference	
Tertile 2	0.66 (0.50, 0.88)	.0043	0.63 (0.47, 0.84)	.0016	0.79 (0.62, 0.93)	<.0001
Tertile 3	0.38 (0.27, 0.53)	<.0001	0.37 (0.27, 0.52)	<.0001	0.83 (0.64, 0.97)	<.0001

Model 1: no covariate was adjusted. Model 2: age, gender, and race were adjusted. Model 3: age, gender, race, education level, income-to-poverty ratio, marital status, insurance status, hypertension, diabetes, and daily energy intake were adjusted.

CI = confidence interval, OBS = oxidative balance score, OR = odds ratio.

### 3.3. Subgroup analyses

To assess the consistency of the correlation between OBS and COPD prevalence among varied populations and identify potential parameters specific to certain groups, subgroup analyses and interaction tests were performed, stratified by age, gender, race, education level, PIR, marital status, and insurance status (Table 4). No significant interactions were observed among any of these stratification variables (*P* for interaction > .05). The inverse relationship between OBS and COPD was more pronounced in individuals without hypertension (OR 0.92, 95% CI 0.90–0.95, *P* < .001). Another variable exhibiting an interaction effect was diabetes. In the population without diabetes, it was observed that for each unit increase in OBS, the risk of COPD decreased by 6% (*P* < .0001). The negative association was not significant in individuals with diabetes.

**Table 4:** Subgroup analysis of the association between oxidative balance score and chronic obstructive pulmonary disease prevalence.

Subgroups	Odds ratio (95% CI)	*P* value	*P* for interaction
Age (yr)			.7811
<60	0.95 (0.92, 0.97)	<.0001	
≥60	0.94 (0.91, 0.97)	<.0001	
Gender			.9623
Male	0.94 (0.91, 0.97)	.0001	
Female	0.94 (0.91, 0.97)	.0003	
Race			.1329
Mexican American	0.91 (0.81, 0.98)	.0025	
Non-Hispanic White	0.94 (0.92, 0.97)	<.0001	
Non-Hispanic Black	0.91 (0.86, 0.97)	.0019	
Others	0.94 (0.89, 0.99)	.0242	
Education level			.0552
Less than high school	0.94 (0.90, 0.98)	.0029	
High school	0.98 (0.95, 1.00)	.0462	
More than high school	0.93 (0.91, 0.96)	<.0001	
PIR			.5870
<1.3	0.93 (0.88, 0.98)	.0037	
1.3–3.5	0.96 (0.89, 0.99)	<.0001	
≥3.5	0.93 (0.90, 0.96)	<.0001	
Marital status			.5451
Married/living with partner	0.94 (0.92, 0.97)	<.0001	
Never married	0.93 (0.90, 0.97)	.0002	
Widowed/divorced/separated	0.96 (0.93, 0.99)	.0015	
Insurance status			.4397
Yes	0.93 (0.90, 0.95)	.0005	
No	0.92 (0.89, 0.94)	<.0001	
Hypertension			.0258
Yes	0.97 (0.94, 0.99)	.0028	
No	0.92 (0.90, 0.95)	<.0001	
Diabetes			.0463
Yes	0.98 (0.96, 1.01)	.2124	
No	0.94 (0.91, 0.97)	<.0001	

Age, gender, race, education level, income-to-poverty ratio, marital status, insurance status, hypertension, diabetes, and daily energy intake were adjusted. In subgroup analyses, the model was not adjusted for the stratification variable itself.

CI = confidence interval, PIR = poverty-to-income ratio.

## 4. Discussion

Unhealthy diet habits and lifestyles are critical factors that result in developing COPD.^[31,32]^ The present study systematically analyzed the correlation between OBS and COPD prevalence in the American population and revealed a significant negative correlation between them. The results suggest that maintaining an antioxidant diet (such as eating foods with high dietary fiber, vitamins, and minerals) and lifestyle (like low alcohol and cotinine intake) will benefit the prevention of COPD.

A Western dietary pattern characterized by higher consumption of pro-oxidative foods such as red meat, cured foods with a high glycemic index, fried food, and margarine was associated with an increased risk of COPD and an accelerated decline of pulmonary function.^[18]^ While previous studies have shown that higher intake of dietary antioxidants is beneficial for lung function, and the negative association between dietary antioxidants and COPD has been widely reported. Studies found that increasing dietary fiber intake may help reduce the risk of developing COPD.^[33,34]^ High dietary fiber has been suggested to attenuate innate immune-mediated systemic and pulmonary inflammation through the presence of a gut–lung axis.^[35]^ Vitamins such as beta-carotene, vitamin C and vitamin E exhibit anti-inflammatory and antioxidant effects and are beneficial for lung function and protective against COPD.^[36,37]^ In addition, minerals may play essential roles in the development of COPD. Studies indicated that dietary zinc, magnesium, and calcium intake were associated with a reduced risk of developing COPD.^[38–40]^ For example, Similar to our study, adopting high-quality and antioxidant-rich nutritional patterns, such as the Mediterranean diet and DASH diet, has been found to exert a protective effect against COPD.^[41,42]^

Lifestyle is believed to be closely related to the occurrence and development of various diseases. Poor lifestyle habits such as lack of exercise, smoking, and excessive alcohol consumption may affect the oxidative stress process,^[43–45]^ leading to excessive reactive oxygen species,^[46,47]^ which in turn damages the structural cells of the airways, leading to airway remodeling and decreased lung function.^[48]^ A multi-cohort study suggested that a normal BMI, never smoking, physical activity, and moderate alcohol consumption were associated with a lower risk for COPD.^[31]^ Additionally, research found that physical activity increased muscle antioxidant content, which might improve muscle function and prevent inflammation and oxidative stress,^[49]^ contributing to a lower risk of COPD.^[50]^ In contrast, a sedentary lifestyle was proved to be associated with a higher prevalence of COPD.^[51]^ In addition, Smoking has been known to be one of the most significant risk factors for COPD.^[47]^ Consistent with previous studies, cigarette smoke is widely known as contributing to COPD. Generated from cigarette smoke, ROS can lead to mitochondrial dysfunction, resulting in increased cell death in lung tissue.^[7]^ Quitting smoking will promote lung function recovery in COPD patients. Moreover, excessive alcohol consumption was found to increase the risk of COPD.^[46]^ Possible mechanisms for alcohol-induced lung injury include increased oxidative stress, altered tissue remodeling, and dysregulated lung inflammation.^[48]^ In recent years, an increasing number of studies have supported that the safe intake of alcohol is no drinking.^[52]^ Furthermore, sustained oxidative stress can activate inflammation-related pathways and trigger the release of inflammatory mediators. The increased presence of proinflammatory mediators attracts more neutrophils and other inflammatory cells, perpetuating lung inflammation in COPD patients.^[7,45]^ Research has found that inflammation levels in obese individuals are significantly higher than those in healthy individuals, suggesting that obesity may also be a risk factor for COPD.^[50]^

In this study, the OBS, which includes 20 oxidative stress factors, was used to quantify dietary and lifestyle-related antioxidant levels. Findings of present study further complement current literature supporting the protective effect of antioxidant-rich dietary patterns and lifestyle habits for COPD, suggesting that dietary and lifestyle interventions may have clinical value in early prevention of COPD.

The results of the subgroup analyses showed statistical differences in the association between OBS and COPD with respect to hypertension and diabetes, which are partially consistent with previous studies. Hypertension and diabetes are strongly associated with increased levels of oxidative stress and higher oxidative stress levels in hypertensive and diabetic populations may diminish the antioxidant capacity of healthy diet and lifestyle habits.^[53,54]^ However, the results of subgroup analyses are only preliminary exploration, and more relevant studies are needed to further validate them.

This survey’s advantage lies in using nationally representative samples from a multiracial population, ensuring representativeness for the general population. Additionally, the study accounted for numerous variables and utilized various statistical methods to improve the reliability of the findings. However, there are limitations to consider. First, its cross-sectional design prevents the establishment or confirmation of causality, and therefore, prospective studies are needed to obtain more conclusive results. Second, reliance on self-reported information may introduce recall bias. Third, the population included in this study was limited to the U.S. population, and more studies are needed to explore the relationship between OBS and COPD in other ethnic. Lastly, while many potential confounders were adjusted for, the impact of different variables cannot be entirely ruled out.

## 5. Conclusions

This study shows a negative relationship between oxidative stress and the odds of COPD, and encouraging antioxidant-rich diets and lifestyles could be crucial in the prevention of COPD. Our research provides a theoretical reference for innovating the clinical management strategies in COPD.

## Acknowledgments

The authors thank all the participants in NHANES study.

## Author contributions

**Conceptualization:** Weiyan Chen, Wei Zhang.

**Data curation:** Weiyan Chen.

**Formal analysis:** Weiyan Chen, Wei Zhang.

**Funding acquisition:** Wei Zhang.

**Investigation:** Weiyan Chen.

**Methodology:** Weiyan Chen.

**Project administration:** Wei Zhang.

**Resources:** Weiyan Chen.

**Software:** Weiyan Chen.

**Supervision:** Wei Zhang.

**Visualization:** Weiyan Chen.

**Writing – original draft:** Weiyan Chen.

**Writing – review & editing:** Wei Zhang.
